# Carcinoma of the oesophagus

**DOI:** 10.1054/bjoc.2000.1766

**Published:** 2001-05

**Authors:** J F Seitz, A Sarradet, E François, J H Jacob, J M Ollivier, P Rougier, A Roussel

**Affiliations:** Institut Paoli-Calmettes, Marseille; Cancer Research Campaign, Paris; Centre Antoine Lacassagne, Nice; Centre François Baclesse, Caen; Hôpital Ambroise Paré, Boulogne, France


					Cancer of the oesophagus has an estimated incidence of 4600-
5600 new cases per year in France and ranks third in frequency
among digestive tract cancers after colon and gastric cancer. It is
responsible for 3.9% of cancer deaths and is the fourth commonest
cause of death after lung, colon, rectum and prostate cancer. The
prognosis is very poor; the French registry shows a 5-year survival
of only 3-6%. These recommendations concern only squamous
(epidermoid) and adenocarcinomas of the oesophagus and none of
the other rarer histological types.
These guidelines were validated in April 2000 and an update is
planned for late 2000.
DIAGNOSIS AND INITIAL ASSESSMENT
The diagnosis of cancer of the oesophagus is based on the
histopathological study of biopsies taken by oesophagogastric
fibroscopy. Staining with Toluidine blue or Lugol can be used to
define more clearly the extent of the primary tumour and/or
demonstrate a second site of disease (defined as a lesion more than
5 cm away from the primary lesion).
Assessment of the extent of disease spread should include (stan-
dard) a complete clinical examination (including nutritional state),
as well as fibreoptic bronchoscopy to exclude the presence of
tracheo-bronchial mucosal extension or a synchronous primary
lesion.
A formal head and neck examination should be done to look for
a synchronous lesion in the oropharynx.
Other assessments include an analysis of respiratory function
(blood gas analysis) as well as cardiological, hepatic and renal
function (standard). Thoraco-abdominal CT scan or abdominal
ultrasound, sub-clavicular ultrasound, oesophagogram and oeso-
phageal echo-endoscopy are all options depending on the results of
initial staging examinations.
CLASSIFICATION
There is no standard for the pre-therapeutic classification of
oesophageal cancers. The us-TNM classification based on echo-
endoscopy is an option. The standard post-surgical classification is
the pTNM classification of the UICC (1997).
TREATMENT MODALITIES
Surgery
The standard technique for curative surgery is a subtotal transtho-
racic oesophagectomy with concurrent nodal clearance and gastro-
plasty if possible.
Surgical treatment is a recommended for stages I and II
(disease localized to the oesophagus). This treatment remains an
option for tumours extending beyond the oesophageal wall
(involving the adventitia (T3) or involving nodes (NI)). Surgery
is not recommended for tumours involving mediastinal organs
(T4) or with distant metastases (M). The inclusion of patients in
therapeutic trials is recommended especially for T3, N0-1
tumours.
Radiotherapy
If chemotherapy is contra-indicated, radiotherapy alone is recom-
mended for the treatment of advanced or inoperable cancers of
the oesophagus. Pre- or postoperative radiotherapy is not recom-
mended for the treatment of oesophageal cancers (option).
Chemotherapy
Chemotherapy for the adjuvant treatment of oesophageal cancer is
not recommended (option, level of evidence B). Therapeutic trials
in which surgery alone is the control should be undertaken.
Chemotherapy for the treatment of advanced oesophageal cancer
is an option, but this must be considered case by case, and be given
when possible within a trial context.
Chemoradiation therapy
Combined modality therapy with radiotherapy and chemotherapy
is superior to radiotherapy alone for the non-surgical treatment of
cancer of the oesophagus (level of evidence B). The RTOG
schedule (four cycles of 5-FU-cisplatin (weeks 1, 5, 8, 11) and
radiotherapy 50 Gy 25 fractions spread over 5 weeks, from week 1
to week 5) can be considered standard treatment for inoperable
cases. It is superior to accelerated high dose per fraction (split
course) radiotherapy (level of evidence B). Combined chemoradi-
ation therapy is an alternative to surgery in operable disease that
penetrates the wall of the oesophagus to involve the adventitia
(T3) or nodes (N1) and also for T1 or T2 tumours (in certain insti-
tutions only). A formal comparison between surgery and chemora-
diation therapy alone has never been undertaken.
Preoperative chemoradiation therapy has not been proven to be
superior to surgery alone in operable subjects with stage I and II
epidermoid cancer of the thoracic oesophagus (level of evidence
B). This treatment remains an option; further trials are necessary.
In operable adenocarcinomas of the oesophagus or the oesopha-
gogastric junction, the combination of chemoradiation therapy is
efficacious when given preoperatively (level of evidence B). This
treatment constitutes a therapeutic option but should still be given
within randomized controlled trials.
Carcinoma of the oesophagus
JF Seitz1
, A Sarradet2
, E Francois3
, JH Jacob4
, JM Ollivier4
, P Rougier5
and A Roussel4
1
Institut Paoli-Calmettes, Marseille; 2
FNCLCC, Paris; 3
Centre Antoine Lacassagne, Nice; 4
Centre Francois Baclesse, Caen; 5
Hopital Ambroise Pare, Boulogne,
France
61
British Journal of Cancer (2001) 84 (Supplement 2), 61-64
(c) 2001 FNCLCC
doi: 10.1054/ bjoc.2001.1766, available online at http://www.idealibrary.com on
Endoscopic therapy
This represents the standard for patients unfit for other treatments.
Formal comparisons with other therapy in prospective trials are
necessary to evaluate the impact on quality of life.
THERAPEUTIC STRATEGY
T1 N0-1 M0, T2 N0-1 M0 disease
There is no standard treatment in operable patients (Figure 1).
Surgical excision (level of evidence C) and combination
62 JF Seitz et al
British Journal of Cancer (2001) 84 (Supplement 2), 61-64 (c) 2001 FNCLCC
Cancer of the oesophagus diagnosed
histologically
T1 or T2 disease
irrespective of N
Patient operable?
Standard
there is no standard
Options
* surgical excision
* combination radiochemotherapy
* adenocarcinoma of the gastro-oesophageal junction:
preoperative combination radiochemotherapy if possible
within a therapeutic trial
Recommendations
surgery for epidermoid carcinomas, preoperative
combination radiochemotherapy within a clinical trial
Standard
combination radiochemotherapy
Option
radiotherapy alone
yes no
Figure 1 Treatment of limited-stage carcinoma of the oesophagus
Histological diagnosis of an oesophageal cancer
T3, N0 -1, M0 T4, N0 -1, M0
Invasion of the tracheal
mucosa?
Standard
there is no standard
Options
* surgery
* combination radiochemotherapy
* radiotherapy alone
* clinical trial
Oesophagopulmonary
fistula?
Standard
there is no standard
options
* palliative surgery
* radiochemotherapy
* therapeutic trial
Standard
oesophageal and/or tracheo-bronchial stent
Standard
there is no standard
Options
* endoscopic treatments
* cautious intermittent hypofractionated
radiotherapy
yes no
yes no
Figure 2 Treatment of advanced stage disease
chemoradiation therapy are options. Surgical resection is recom-
mended. Preoperative chemoradiation therapy must be re-
evaluated within therapeutic trials. For patients with inoperable
disease, chemoradiation therapy is the standard treatment. If
chemotherapy is contra-indicated, radiotherapy alone is a thera-
peutic option.
US T3 N0-1 M0 disease
There is no standard. Surgical excision, chemoradiation therapy
(or radiotherapy alone if chemotherapy is contra-indicated) and
chemoradiation therapy followed by surgery are the therapeutic
options (Figure 2). Patients should be included in prospective
randomized trials.
US T4 N0 and T4 N1 disease without invasion of the
tracheal mucosa
There is no standard. Palliative surgery, chemoradiation therapy or
radiotherapy alone (if chemotherapy is contra-indicated) are
options (Figure 2). Patients should be included in prospective
randomised trials.
US T4 N0 and T4 N1 disease with involvement of the
tracheal mucosa
a) Patients without oesophageal-respiratory tract fistulae. There is
no standard. Endoscopic treatments for dysphagia and respiratory
compromise or radiotherapy (using small doses per fraction) with
or without chemotherapy for patients of reasonable performance
status are therapeutic options.
b) Patients with oesophageal-respiratory tract fistulae. The place-
ment of an oesophageal and/or tracheo-bronchial stent constitutes
standard treatment.
Metastatic oesophageal cancer
a) Coeliac axis and cervical node metastases (M1). There is no stan-
dard treatment. Chemotherapy, combination chemoradiation therapy
and endoscopic treatments are therapeutic options. (Figure 3)
b) Visceral metastases. If the primary oesophageal tumour has not
been resected, and if the patient is of good performance status (PS1
or 2), there is no standard. Combination chemoradiation therapy
followed by chemotherapy alone (if there is evidence of an
objective response), endoscopic treatment for dysphagia and/or
chemotherapy are therapeutic options. If the oesophageal tumour
has not been resected and if the patient is of poor performance
Carcinoma of the oesophagus 63
British Journal of Cancer (2001) 84 (Supplement 2), 61-64
(c) 2001 FNCLCC
Metastatic cancer of the oesophagus
Nodal metastases (M1) Visceral metastases
Unresected
oesophageal tumour?
Standard
there is no standard
Options
* endoscopic treatments
* combination
radiochemotherapy
* chemotherapy and treatment of
dysphagia
PS1 or 2 PS3 or 4 PS1 or 2 PS3 or 4
Standard
there is no standard
Options
* endoscopic treatment
* combination radiochemotherapy
* chemotherapy and treatment of
dysphagia
Standard
endoscopic treatment
of dysphagia
Standard
there is no standard
Option
chemotherapy (clinical trial)
Standard
treatment of symptoms
yes no
Figure 3 Treatment of metastatic disease
status (PS3 or 4) the standard therapy is endoscopic therapy for the
palliation of dysphagia.
If the primary oesophageal tumour has been resected and if the
patient has a good performance status (PS1 or 2) there is no stan-
dard. Chemotherapy, preferably within the context of a therapeutic
trial is an option.
If the oesophageal tumour has been resected and if the patient
is of poor performance status, symptomatic treatment is stan-
dard.
Non-metastatic adenocarcinoma of the cardio-
oesophageal junction
If the patient has operable disease, surgical excision is standard.
Preoperative chemoradiation therapy, preferably within a clinical
trial is a therapeutic option. If the patient does not have operable
disease, combination chemoradiation therapy is standard. When
chemotherapy is contra-indicated, radiotherapy alone and/or endo-
scopic treatments are therapeutic options.
FOLLOW-UP
The follow-up of patients with oesophageal cancer relies on clin-
ical examination focusing on dysphagia, nutritional status and the
sites of likely nodal relapse. There is no consensus as to the
interval between assessments (3-6 months). Paraclinical follow-
up (i.e. by imaging and blood tests) is an option, especially if
patients are entered into therapeutic trials. A formal head and neck
examination should be undertaken 12-18 months after initial treat-
ment in patients without recurrence.
INTERNAL REVIEWERS
T Conroy (Centre Alexis Vautrin, Vandoeuvre-Les-Nancy), M
Ducreux (Institut Gustave Roussy, Villejuif), D Elias (Institut
Gustave Roussy, Villejuif), D Froissart (Institut Jean Godinot,
Reims), P Haegele (Centre Paul Strauss, Strasbourg), JM
Hannoun-Levi (Institut Paoli Calmettes, Marseille), P Houyau
(Institut Claudius Regaud, Toulouse), JH Jacob (Centre Francois
Leclerc, Dijon), G Monges (Institut Paoli Calmettes, Marseille), S
Nasca (Institut Jean Godinot, Reims), TD Nguyen (Institut Jean
Godinot, Reims), JM Ollivier (Centre Francois Baclesse, Caen), B
Paillot (Centre Henri Becquerel, Rouen), JL Raoul (Centre Eugene
Marquis, Rennes), JF Seitz (Institut Paoli Calmettes, Marseille), J
Stines (Centre Alexis Vautrin, Vandoeuvre-Les-Nancy), P
Troufleau (Centre Alexis Vautrin, Vandoeuvre-Les-Nancy).
EXTERNAL REVIEWERS
L Bedenne (CHRU-Hopital General, Dijon), JF Bosset (CHU,
Besancon), PM Bret (The Montreal General Hospital, Montreal),
G Calais (Hopital Bretonneau, Tours), E Calitchi (Boulogne), R
Coquard (Clinique Saint-Jean, Lyon), J Desbaumes (Hopital
d'Instruction des Armees Desgenettes, Lyon), PL Etienne
(Clinique Armoricaine, Saint-Brieuc), G Ganem (Centre Jean
Bernard, Le Mans), JP Gerard (Centre Hospitalier Lyon Sud,
Pierre Benite), H Gouerou (Hopital Augustin Morvan, Brest), S
Greget (Clinique Sainte-Clotilde, Sainte-Clotilde), D Langlois
(Centre Saint-Michel, La Rochelle), B Launois (CHU
Pontchaillou, Rennes), S Naveau (Hopital Antoine Beclere,
Clamart), R Soleilhac (Clinique Sainte-Clotilde, Sainte-Clotilde),
JP Triboulet (CHG, Lille), M Untereiner (Hopital Claude Bernard,
Metz), JF Velly (Hopital du Haut-Leveque, Pessac).
64 JF Seitz et al
British Journal of Cancer (2001) 84 (Supplement 2), 61-64 (c) 2001 FNCLCC

				

